# Radicular Cyst Presenting in a Female Child: A Case Report

**DOI:** 10.7759/cureus.45872

**Published:** 2023-09-24

**Authors:** Anvika Deshpande, Amit Reche, Mihika Deshpande, Shreyash Borkar, Samruddhi Rathi

**Affiliations:** 1 Public Health Dentistry, Sharad Pawar Dental College, Datta Meghe Institute of Higher Education and Research, Wardha, IND

**Keywords:** inflammation, sinus, curettage, epithelium, apical periodontal cyst

## Abstract

A radicular cyst, also known as a periapical cyst or root end cyst, is usually associated with permanent dentition and its association with deciduous teeth is an infrequent phenomenon. Radicular cyst typically arises from inflammation of the pulp and periapical tissues of a non-vital tooth. This article presents a case report of a six-year-old female with an apical periodontal cyst associated with deciduous and permanent teeth in the maxillary posterior region of the jaw. Early diagnosis of the lesion might have resulted in a conservative treatment plan. This text's purpose is to emphasize the pedodontist’s role in the early analysis of such lesions.

## Introduction

Apical periodontal cyst is a very rare phenomenon in deciduous dentition with a prevalence of only 0.5% to 3.3% of all the recorded jaw cysts [1,2]. A radicular cyst is an inflammatory odontogenic cyst that is mainly derived from inflammatory stimulation to epithelial cell rests of malassez which are present along the periodontal ligament area of the tooth which usually is a sequela of bacterial infection and dental pulp necrosis. The majority of cases are asymptomatic and present with no clinical evidence of their presence and are accidentally found on the radiograph on routine general examination and present with no clinical evidence of their presence [3]. The most commonly affected jaw is the maxilla then the mandible and tooth affected in the former are maxillary anterior mainly central incisors which are followed by mandibular posterior mainly first molar. The lesion is almost always apical but can sometimes be found laterally. Deciduous dentition mainly affects the mandibular jaw whereas chances of permanent dentition affecting the maxilla are higher [4]. The epithelial lining of the radicular cyst may sometimes depend on the location of the cyst, for example, the periapical lesion may be lined by respiratory epithelium if it contacts the maxillary sinus [3]. The radiographic findings include a radiolucent area with radio-opaque borders associated with the apical region of the involved tooth. The treatment includes conventional nonsurgical root canal therapy or extraction if the tooth cannot be preserved due to carious involvement and periapical surgery followed by curettage. Very rarely can the treated radicular cyst give rise to residual cyst [4].

## Case presentation

A six-year-old patient came to the outpatient department of Oral Medicine and Radiology with a complaint of pain in the upper right posterior region of the jaw for one month and swelling in the same region extra-orally for 10 days. The patient’s parents gave a history of root canal treatment with the lower left posterior tooth and the past dental history revealed extraction of the tooth in the upper right posterior region of the jaw two years ago but clinical and radiological examination do not confirm the same. The history of the present illness revealed that the patient was alright one month back then he experienced pain in the upper right posterior region of the jaw. The pain was severe sharp shooting, intermittent. The pain was aggravated by chewing food and relived by taking medications. The swelling was present in the upper right molar region which gradually increased and reached the present size. There was a history of fever for three days, a history of paraesthesia over the region, and discomfort while bending down for eight days. The patient did not give any history of increase or decrease in the size of swelling and there was no history of bleeding or pus discharge.

**Figure 1:**
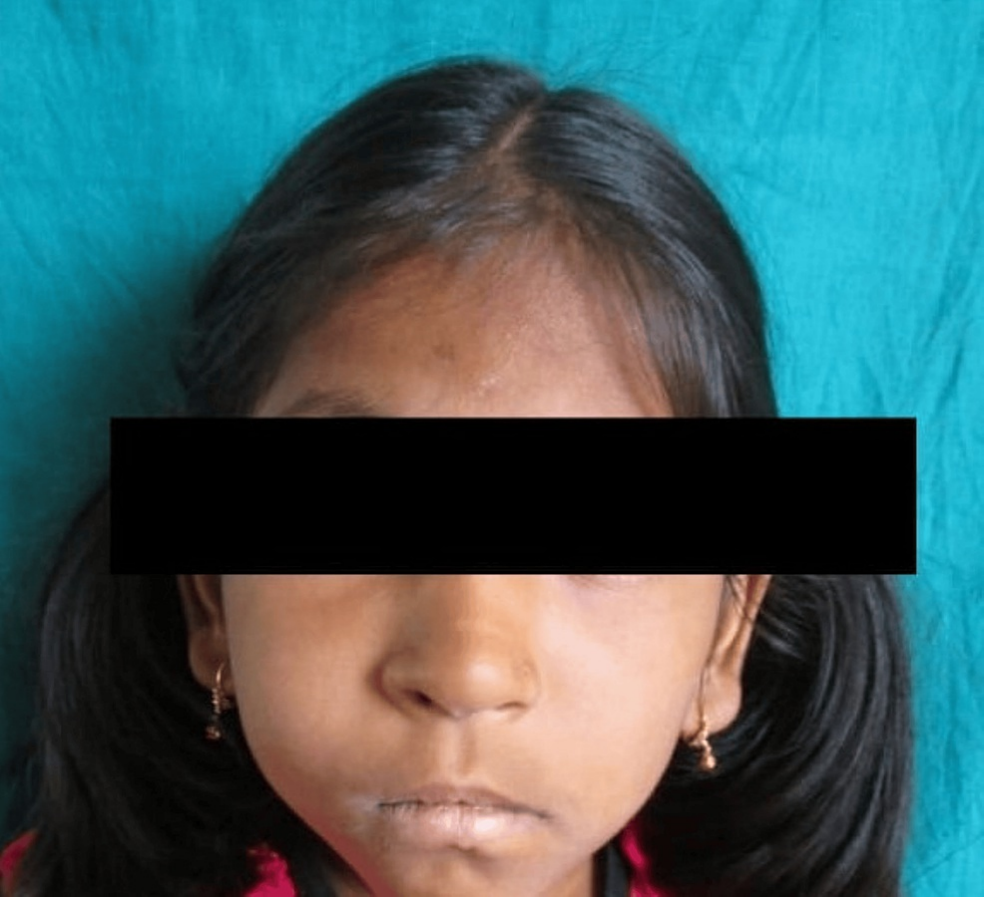
Extraoral view showing diffuse swelling over right side of face.

On extraoral examination, the face was asymmetrical due to swelling present on the upper right malar region (Figure 1) extending anteroposteriorly from ala of the nose to the line joining the outer canthus of the eye.

**Figure 2:**
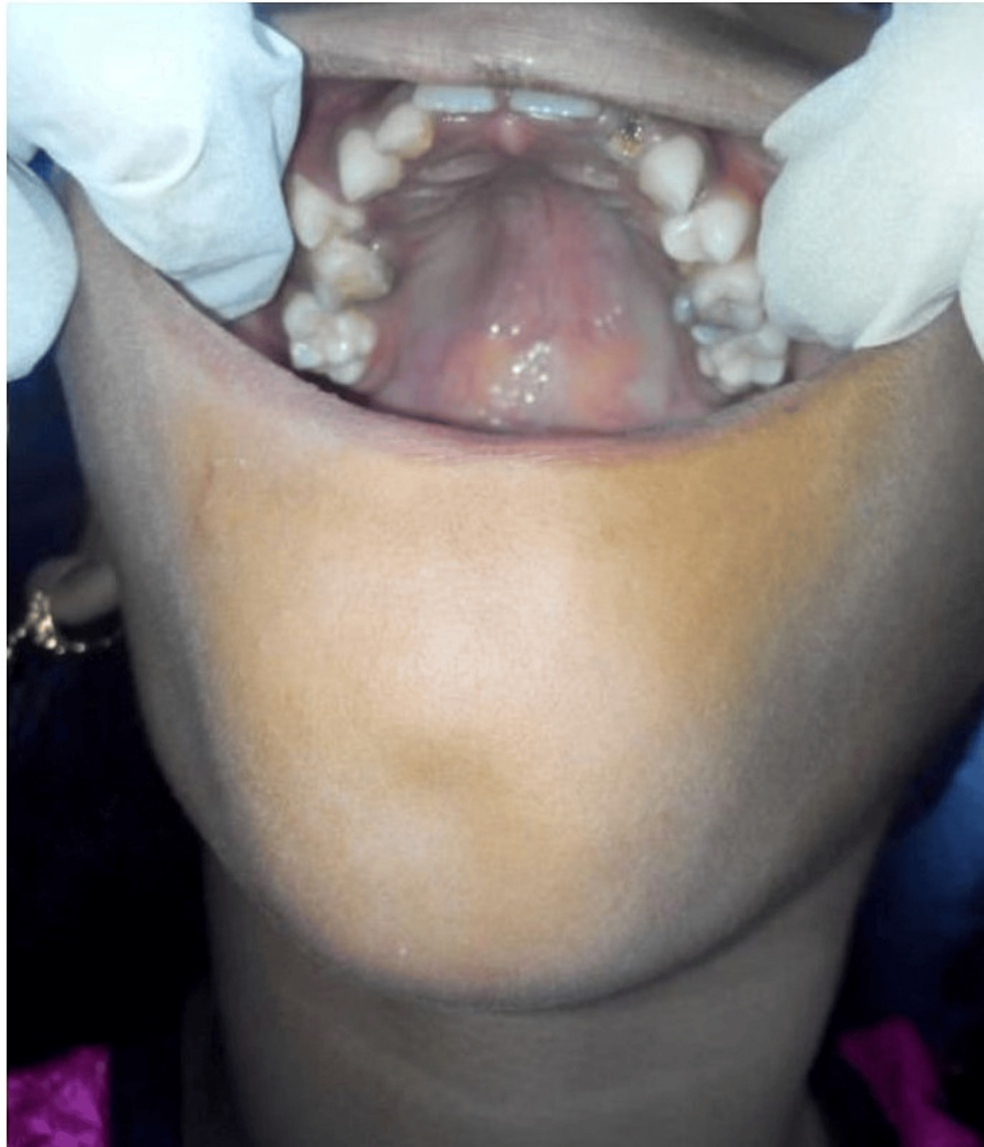
Intraoral view showing diffuse swelling in upper right posterior region of jaw.

Supero-inferiorly from right infraorbital ridge region till the level of corner of the mouth of size 3x3 cms approximately, shape roughly round, surface smooth, margins were diffuse. On palpation, the temperature was raised, the swelling was fixed to underlying structures and the consistency was soft. On intraoral examination, approximately a single swelling was seen in the upper right posterior region associated with 55, 16 of size 3x2 cm (Figure 2).

The shape was roughly oval with a smooth surface, diffused margins, soft consistency, and absent tenderness. There were pit caries with 55 and grade III mobility with 53, stains and calculus were also found.

**Figure 3:**
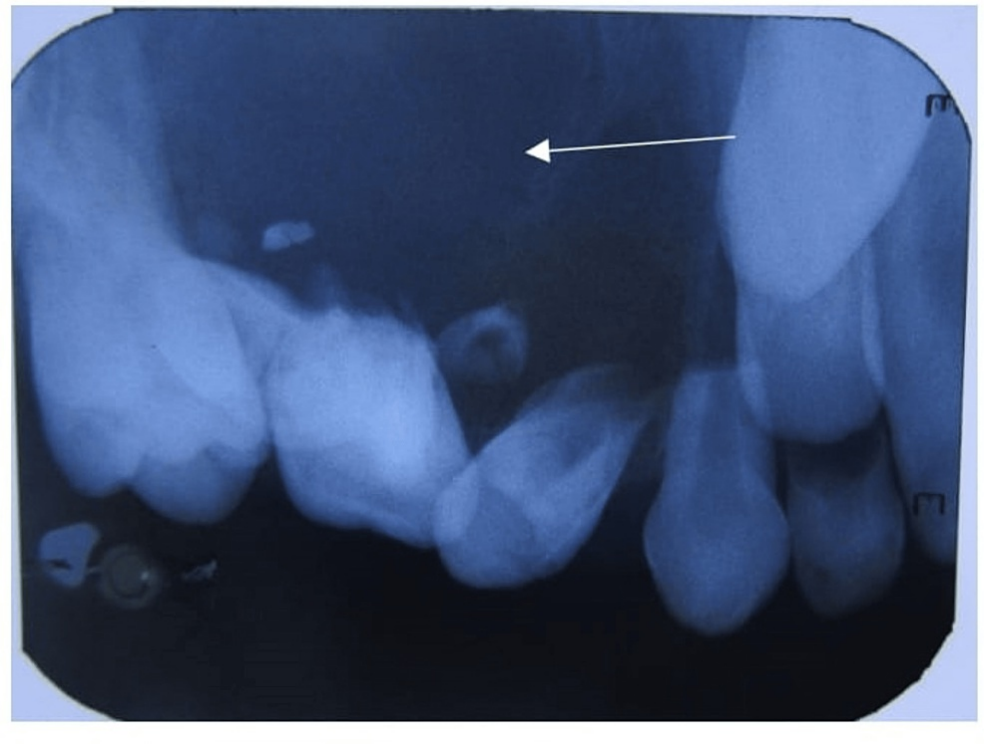
An intra-oral periapical radiograph revealing a radiolucency along the roots of the deciduous second molar and permanent first molar.

Given all the clinical features, just like a few normally occurring lesions inside the oral cavity, the provisional diagnosis of acute exacerbation of chronic periapical abscess with 55 and 16 was made, radicular cysts with 55 and 16 and chronic periapical abscess with 55 were kept in the differential diagnosis. As a part of investigations an intra-oral periapical (IOPA) radiograph was advised with 55 and 16 regions, taking into consideration the size of the lesion maxillary occlusal, and panoramic radiographs were advised. The radiographs revealed maxillary right permanent premolars were erupted and their roots were yet to complete development. An intra-oral periapical radiograph revealed a radiolucency along the roots of the deciduous second molar and permanent first molar and developing premolars depicted by the calcifications in the radiography (Figure 3).

**Figure 4:**
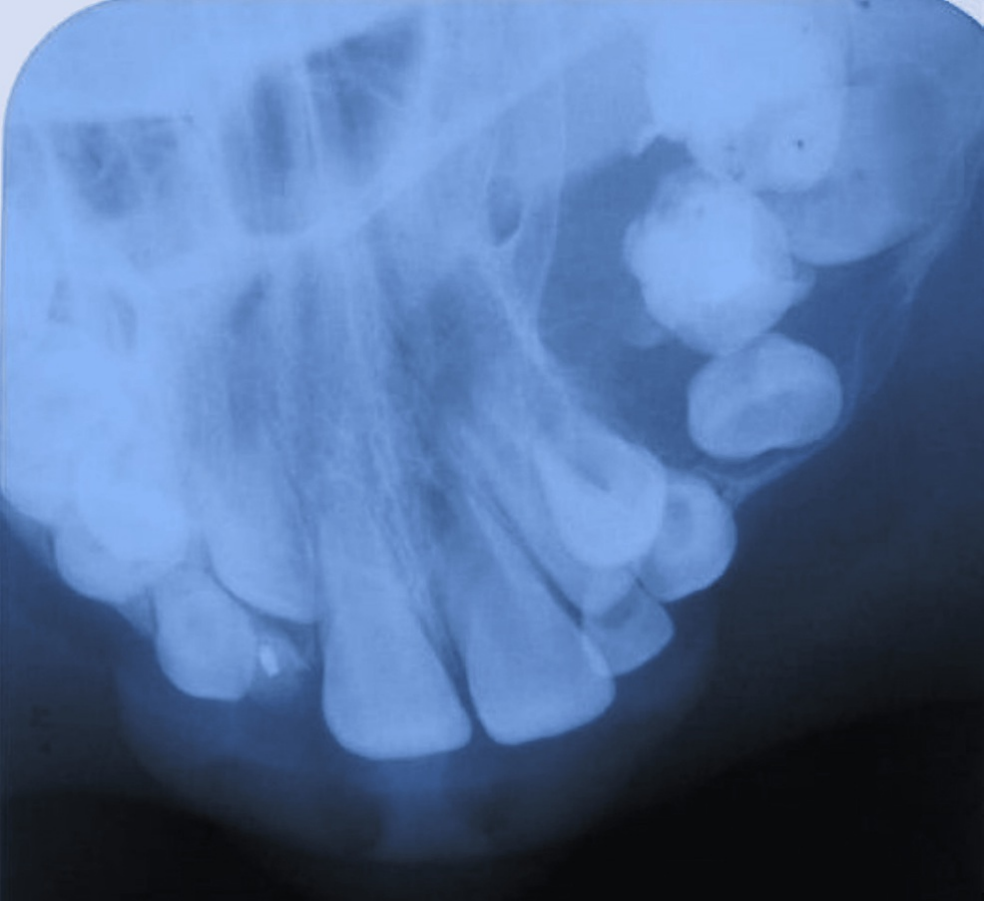
The maxillary right occlusal radiograph revealed an unusual, well-defined bilocular radiolucency with the expansion of both lingual and buccal cortical plates.

The maxillary occlusal radiograph revealed an unusual, well-defined bilocular radiolucency with the expansion of both lingual and buccal cortical plates (Figure 4).

**Figure 5:**
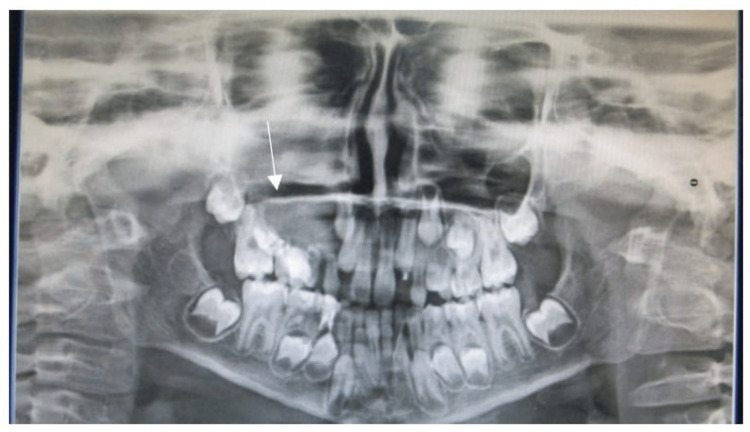
Panoramic view shows a well-defined radiolucency seen in periapical region of 54, 45, 16 region of size 2x3 cm approximately, roughly oval in shape, with well-corticated borders.

The panoramic radiograph showed a single well-defined radiolucency in the periapical region of 54, 55 and 16 regions of size 2x3 cm approximately, roughly oval, with well-corticated borders (Figure 5).

**Figure 6:**
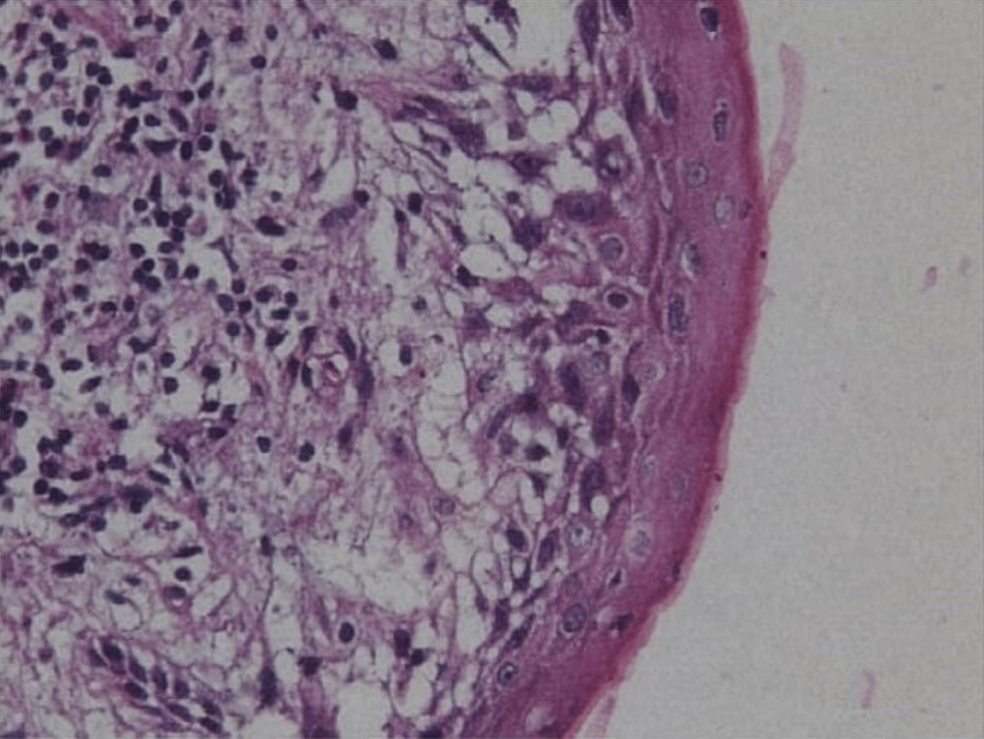
Histopathological examination shows epithelium with underlying connective tissue containing numerous inflammatory cell infiltrates.

All the radiographic findings were suggestive of radicular cysts with 54, 55, and 16, and an incisional biopsy was advised. After taking the incisional biopsy, the histological examination showed epithelium with underlying connective tissue containing numerous inflammatory cell infiltrates (Figure 6).

The lesion was surgically enucleated, the specimen was sent for histopathological evaluation and the follow-up was ensured. In the follow-up visit the patient's extraoral health had improved and was bilaterally symmetrical, lips were competent and lymphadenopathy was absent. Intraorally no abnormalities were detected hence no postoperative radiograph was taken.

## Discussion

A radicular cyst also known as a periapical cyst or dental cyst is more predominant in males than females. Its incidence in maxillary dentition is more than mandibular dentition. This is the most common of all cysts occurring in the jaw and is derived from cell rests of Malassez situated around the roots of the teeth; they often go unnoticed and are especially neglected in primary dentition due to their rare occurrence. They are expected to resolve once the primary tooth falls off or is extracted and thereby is left untreated. The radicular cysts are usually asymptomatic unless secondarily infected [4,5]. In the present case report, there was a radicular cyst associated with deciduous as well as permanent teeth in the maxillary posterior region of the jaw in a six-year-old female. Commonly encountered odontogenic lesions are periapical abscesses, radicular cysts, dentigerous cysts, ameloblastoma, and odontogenic keratocyst. Mostly, multilocular lesions favor the mandibular posterior region and those showing expansion is considered ameloblastoma and their variants depending on the age and their presenting characteristics whereas unilocular lesions are labeled as odontogenic cyst both clinically and radiographically [6-8].

Grundy et al. described a series of radicular cysts associated with deciduous teeth that were treated endodontically with material containing formocresol, which is antigenic and has been demonstrated to trigger a humoral and cell-mediated immune response when combined with tissue protein. In the present case, a radicular cyst of size 2x3 cm along with palatal expansion was seen. However, a radicular cyst rarely exceeds 1 cm in size, but if larger it might show buccal or lingual cortical plate expansion and can thin the bone around the tooth [4]. Although radicular cysts in primary teeth are rare, in the present case it was seen in a six-year-old child. In a wide-ranging survey of 1300 periapical cysts with deciduous and permanent dentition, the prevalence of radicular cysts associated with primary teeth was only 0.5% [9]. Odontogenic keratocyst can be given as a differential diagnosis in occasions of aggressive, large osteolytic lesions of the jaw. In that case, genetic testing and family history are required to assess nevoid basal cell carcinoma syndrome for this age. Prompt diagnosis and treatment ensure the great success of the procedure [10]. 

Surgical treatment of apical periodontal cysts is almost always enucleation and it is mostly recommended that the permanent teeth associated with the lesion should be preserved whereas the preservation of deciduous teeth may vary according to the situation. Other most commonly performed surgeries are marsupialization (Partsch method), enucleation with primary packing, or marsupialization followed by enucleation (Waldron’s technique). Based on the surgeon's point of view and assessment of all the necessary findings a clinical diagnosis of the radicular cyst with 55, 16 was confirmed. It was decided to surgically incise the lesion under general anesthesia and then suture using nonabsorbable silk sutures. The patient was then recalled after seven days for removal of the sutures and hence granulation tissue was successfully formed and the wound healed by primary intention. Treating patients with apical periodontal cysts in deciduous dentition is a tedious task and hence needs a lot of patience and patient compliance for the treatment to be successful and have a full-term recovery. Similar studies have been successfully done at my university [11,12].

## Conclusions

In conclusion, a radicular cyst is a common condition affecting the oral cavity. It remains unnoticed most of the time and rarely reaches the tactile dimension. In this unusual case report, although a large lesion involving the primary and permanent teeth the most commonly occurring lesion like a radicular cyst should be kept in the provisional diagnosis. This case depicts a common ailment that affects a unique age group and site. Especially in cases of deciduous dentition, the cystic potential should not be ignored even though the healing of post-surgical osseous deformities in children is always good since they have a high proclivity for bone regeneration.
